# Correlation between prediabetes and coronary artery disease severity in patients undergoing elective coronary angiography

**DOI:** 10.1186/s43044-019-0034-y

**Published:** 2019-12-27

**Authors:** Ahmed Muhammed, Mohamed Tarek Zaki, Ahmed Shawky Elserafy, Sameh Attia Amin

**Affiliations:** Department of Cardiology, Ain Shams University, Cairo, Egypt

**Keywords:** Prediabetes, Coronary artery disease, Elective coronary angiography

## Abstract

**Background:**

Diabetes is a chronic disease that is responsible for a high rate of morbidity and mortality which can be attributed to atherosclerosis and cardiovascular disease. Diabetes is heralded by prediabetes which not only indicates a higher risk of developing diabetes but also increases the burden of cardiovascular disease. The objective was to observe the effect of prediabetes on the severity of coronary artery disease in patients undergoing elective coronary angiography. Seven hundred and thirty-one patients were admitted for elective coronary angiography and/or PCI starting from September 2017 to August 2018. Patients were divided into group A (normoglycemic group, *N* = 228), group B (prediabetes group, *N* = 177), and group C (diabetic group, *N* = 326). Coronary artery disease (CAD) severity including number of vessels affected and atherosclerotic burden by Gensini score were compared among different groups.

**Results:**

The number of vessels affected as well as left main (LM) disease was higher in the prediabetes group when compared to the normoglycemic group (P,=0.001, *P* = 0.009, respectively) and was comparable to the diabetes group (*P* = 0.4, *P* = 0.6, respectively). Prediabetes showed a Gensini score higher than the normoglycemic group (*P* = 0.0001) with no significant difference when compared to the diabetic group (*P* = 0.9).

**Conclusion:**

Prediabetes is associated with high atherosclerotic burden and coronary artery disease complexity that is similar to diabetic than normoglycemic individuals.

## Background

Diabetes is a chronic disease that is responsible for high rates of morbidity and mortality which can be attributed to atherosclerosis and cardiovascular disease [1]. It is estimated that type II diabetes doubles the risk of cardiovascular disease even after adjustment of other cardiovascular risk factors [2]. Despite the increase in the rate of treatment of diabetic patients with statins and glucose lowering drugs achieving target glycated hemoglobin (HBA1C) levels and low-density lipoprotein (LDL) levels [3], another strategy of effective management of diabetes lies in management of the disease process at earlier stage [1]. Prediabetes is a collective term that encloses individuals with glucose levels lower than cutoff levels for diabetes but too high to be considered normal. It is the term used for individuals with impaired fasting glucose (IFG) and/or impaired glucose tolerance (IGT) and/or HbA1C levels ranging from 5.7 to 6.4% [3]. Prediabetes is not an uncommon condition with an estimated worldwide prevalence of 343 million individuals expected to rise to 471 million by 2035 [4]. Prediabetes is a serious clinical condition that not only increases the risk of developing diabetes but also increases the burden of cardiovascular disease risk. Compared to normoglycemic individuals, patients with prediabetes show a 20% higher risk of developing cardiovascular disease (CVD) [5]. Prediabetes is a toxic state in which both micro- and macrovascular complications of diabetes can manifest [6]. The prompt diagnosis and proper management of prediabetes are necessary to prevent progression to diabetes mellitus and to prevent microvascular and macrovascular complications that manifest early in the prediabetic state [7]***.***

## Aim of the work

Observes the effect of prediabetes on the severity of coronary artery disease in patients undergoing elective coronary angiography.

## Methods

The current study was carried out at the cardiology department at a university hospital.

### Inclusion criteria

Patients who were admitted for elective coronary angiography and/or PCI starting from September 2017 to August 2018.

### Exclusion criteria

No exclusion criteria were applied.

After an informed written consent, all patients involved in the study were subjected to:

**A- History taking and examination** with special emphasis on age, sex, risk factors for coronary artery disease (smoking, HTN, DM, dyslipidemia, positive family history for premature CVDs), history of CKD detected either by reduction in GFR or high serum creatinine, history of prior percutaneous coronary intervention (PCI) or coronary arteries bypass grafting (CABG), or acute coronary syndrome (ACS).

**B- Laboratory tests**: Level of HBA1C and serum creatinine on admission.

**C- Estimation of renal function**: eGFR was estimated using MDRD formula:

eGFR = 186 × (serum creatinine)–1.154 × age–0.0203 (× 1.210 if black) (× 0.742 if female) [8]

**D- Interventional data**: Number of vessels affected and atherosclerotic burden of CAD assessed by Gensini score [9]***.*** For patients undergoing PCI, additional data was collected regarding number of stents used, type of stents used, and total length of stents used.

The studied patients were divided according to HbA1C level to 3 groups:

1- Group A: Normoglycemic patients (HBA1C < 5.7%)

2- Group B: Prediabetic patients (HBA1C 5.7–6.4%)

3- Group C: Diabetic patients (HBA1C > 6.4%) [10]

### Statistical analysis

Data were collected and revised on PC. Data were tabulated and statistically analyzed using SPSS 17 software, mean and standard deviation (± SD), and range for parametric numerical data, while the median was used for nonparametric numerical data. Student t-test was used to assess the statistical significance of the difference between two study group means. Mann–Whitney test (U test) was used to assess the statistical significance of the difference of a nonparametric variable between two study groups. Chi-squared test was used to examine the relationship between two qualitative variables. Fisher’s exact test was used to examine the relationship between two qualitative variables when the expected count is less than 5 in more than 20% of cells.

## Results

Patients were divided to group A (normoglycemic group, *N* = 228), group B (prediabetes group, *N* = 177), and group C (diabetic group, *N* = 326). Prediabetics represented 24% of the study population (Table 1).

**Table 1:** Group classification

HBA1C	Group A(*n* = 228) (31.2%)	Group B(*n* = 177) (24.2%)	Group C(*n* = 326) (44.6%)
Mean ± SD	5.25 ± 0.24	6.00 ± 0.22	8.92 ± 1.60
Range	4.5–5.6	5.7–6.4	6.5–13

Among patients with HBA1C in the prediabetic range, there were only 8 patients who were known prediabetic and on medical treatment. Among the diabetic group, 7% of patients were newly diagnosed, denoting that newly diagnosed prediabetics and diabetics represent 26% of the study population.

### Demographic and clinical characteristics

There was no significant difference regarding age among the three groups, yet group C showed higher prevalence of male gender and a lower prevalence of smoking. Both DM and prediabetes group showed significantly higher prevalence of HTN. The normoglycemic group showed a stronger family history of CAD (Table 2).

**Table 2:** Demographic and clinical characteristics of the groups

	Group A	Group B	Group C	Test value	*P* value	Sig.	Post hoc analysis		
	No. = 228	No. = 177	No. = 326				P1	P2	P3
Age (years)	56.68 ± 9.21	57.10 ± 9.84	58.26 ± 8.87	2.166	0.115	NS	–	–	–
Sex(male)	171 (75.0%)	132 (74.6%)	213 (65.3%)	7.823	0.020	S	0.924	0.015	0.033
Smoking	114 (50.0%)	99 (55.9%)	111 (34.0%)	26.588	0.000	S	0.236	0.000	0.000
HTN	84 (36.8%)	90 (50.8%)	198 (60.7%)	30.649	0.000	S	0.005	0.000	0.032
Dyslipidemia	120 (52.6%)	86 (49.0%)	165 (50.6%)	0.66	0.7	NS	–	–	–
Known CKD	12 (5.3%)	9 (5.1%)	10 (3.1%)	2.002	0.367	NS	–	–	–
Family history of CAD	97 (42.5%)	36 (20.3%)	82 (25.2%)	28.804	0.000	S	0.000	0.000	0.224

*P* value > 0.05, nonsignificant; *P* value < 0.05, significant; *P* value < 0.01, highly significant

*: Chi-squared test; •: one-way ANOVA test

P1: *P* value group A vs group B

P2: *P* value group A vs group C

P3: *P* value group B vs group C

### Assessment of renal function

On comparing the three groups, there was no significant difference regarding the mean eGFR or prevalence of CKD (Table 3).

**Table 3:** Assessment of renal function

		Group A	Group B	Group C	Test value	*P* value	Sig.
		No. = 228	No. = 177	No. = 326			
Creatinine	Mean ± SD	1.03 ± 0.26	1.05 ± 0.25	1.02 ± 0.29	0.980•	0.376	NS
	Range	0.6–1.6	0.6–1.9	0.5–2.9			
eGFR	Mean ± SD	78.86 ± 23.31	76.92 ± 23.28	78.41 ± 24.12	0.361•	0.697	NS
	Range	35–142	37–142	25–149			
CKD		54 (23.7%)	33 (18.6%)	75 (23.0%)	1.711*	0.425	NS

*P* value > 0.05, nonsignificant; *P* value < 0.05, significant; *P* value < 0.01, highly significant

*, Chi-squared test; •, one-way ANOVA test

P1: Group A vs group B

P2: Group A vs group C

P3: Group B vs group C

### Prior history of ischemia

There was no significant difference in history of PCI or CABG prior to the current procedure between the different groups with significantly higher prevalence of prior ACS in patients with prediabetes (Table 4).

**Table 4:** History of CAD among the different groups

	Group A	Group B	Group C	Test value*	*P* value	Sig.	Post hoc analysis		
	No. (%)	No. (%)	No. (%)				P1	P2	P3
Prior PCI	42 (18.4%)	39 (22.0%)	75 (23.0%)	1.747	0.417	NS	–	–	–
Prior CABG	9 (3.9%)	3 (1.7%)	15 (4.6%)	2.784	0.249	NS	–	–	–
Prior ACS	81 (36.0%)	93 (52.5%)	114 (35.0%)	16.543	0.000	S	0.001	0.803	0.000

*P* value > 0.05, nonsignificant; *P* value < 0.05, significant; *P* value < 0.01, highly significant

*: Chi-squared test

P1: Group A vs group B

P2: Group A vs group C

P3: Group B vs group C

### Interventional data

**Fig. 1:**
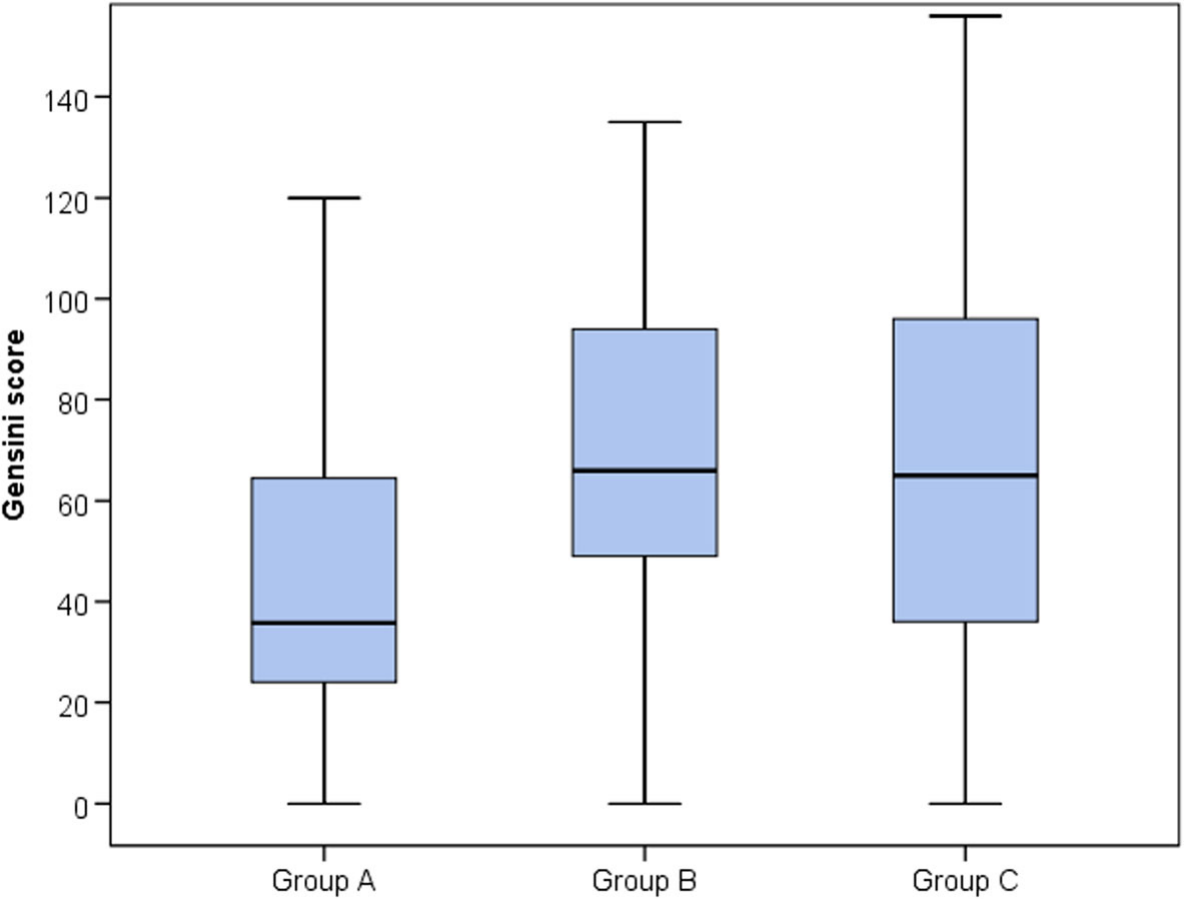
CAD severity among different groups represented by Gensini score (median and IQR)

**Fig. 2:**
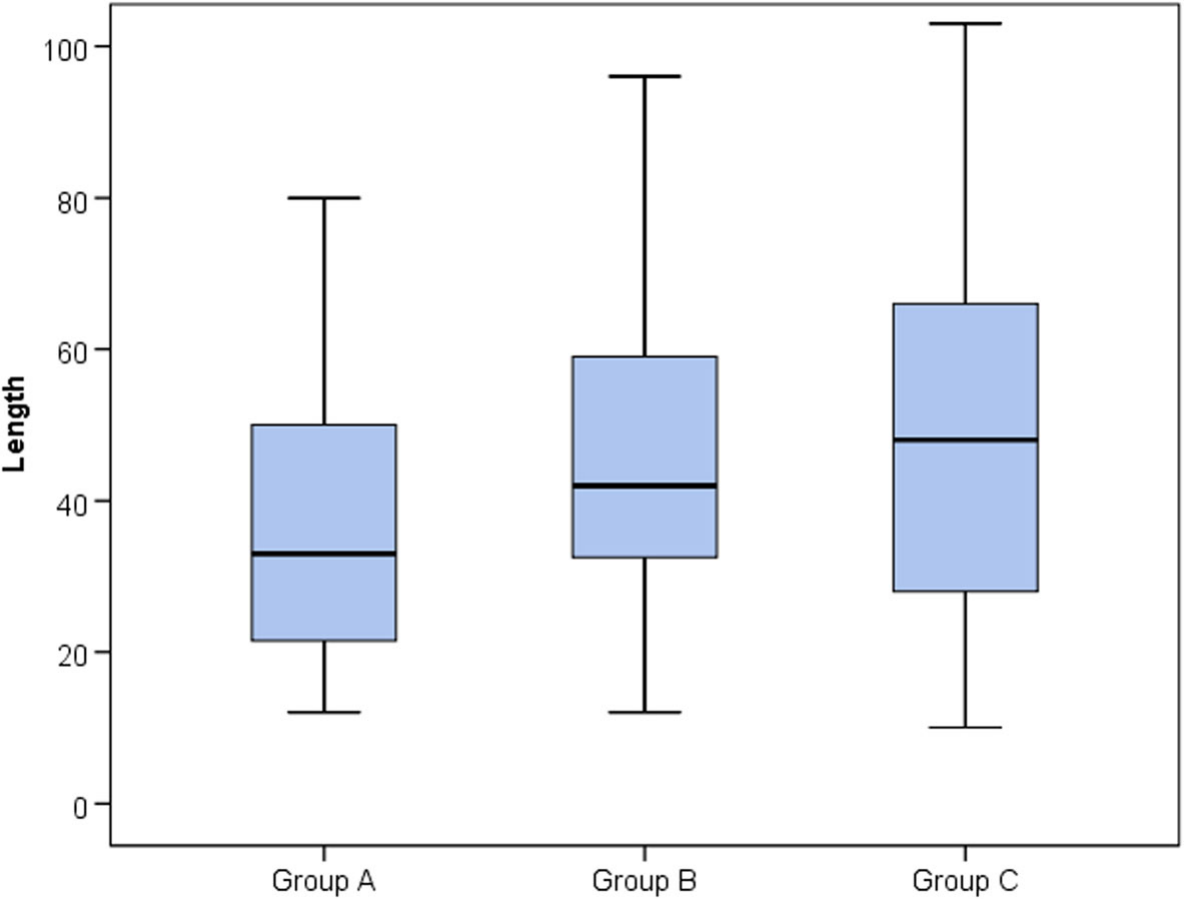
Length of stents used among different groups

Regarding the type of procedure performed, group A showed a lower rate of PCI compared to group C. Both group B and group C showed a larger number of vessels with significant disease when compared to group A. LM disease was significantly higher in groups B and C when compared to group A. Group B showed a more complex coronary anatomy with a higher Gensini score than group A and comparable to group C. The type of stent used was similar among the different groups. Length of stents used was higher in prediabetic when compared to normoglycemic group denoting a longer length of lesions (Table 5, Figs. 1 and 2).

**Table 5:** Interventional data among the different groups

		Group A	Group B	Group C	Test value	*P* value	Sig.	Post hoc analysis		
		No. = 228	No. = 177	No. = 326				P1	P2	P3
Procedure	CA	99 (43.4%)	66 (37.3%)	116 (35.6%)	14.507*	0.024	S	0.215	0.009	0.662
	CA + PCI	69 (30.3%)	57 (32.2%)	93 (28.5%)						
	CA + PTCA	3 (1.3%)	0 (0.0%)	0 (0.0%)						
	PCI	57 (25.0%)	54 (30.5%)	117 (35.9%)						
No. of vessels	0	42 (18.4%)	18 (10.2%)	38 (11.7%)	41.574*	0.000	S	0.000	0.000	0.435
	1	102 (44.7%)	54 (30.5%)	87 (26.7%)						
	2	48 (21.1%)	66 (37.3%)	111 (34.0%)						
	3	36 (15.8%)	39 (22.0%)	87 (26.7%)						
	4	0 (0.0%)	0 (0.0%)	3 (0.9%)						
LM disease		11(4.8%)	21(11.8)%	34(10.4%))	7.418	0.0245	S	0.009	0.01	0.6
Gensini score	Median (IQR)	35.75 (24–64.5)	66 (49–94)	65 (36–96)	72.404≠	0.000	S	0.000	0.000	0.967
	Range	0–152	0–135	0–156						
Type of stent	No	0 (0.0%)	3 (2.7%)	3 (1.4%)	6.393*	0.172	NS	–	–	–
	BMS	0 (0.0%)	3 (2.7%)	3 (1.4%)						
	DES	120 (100.0%)	105 (94.6%)	204 (97.1%)						
No. of stents	0	3 (2.4%)	3 (2.7%)	3 (1.4%)	22.963*	0.003	S	0.103	0.000	0.331
	1	78 (63.4%)	54 (48.6%)	90 (42.9%)						
	2	39 (31.7%)	48 (43.2%)	99 (47.1%)						
	3	0 (0.0%)	3 (2.7%)	15 (7.1%)						
	4	3 (2.4%)	3 (2.7%)	3 (1.4%)						
Length	Median (IQR)	33 (21.5–50)	42 (32.5–59)	48 (28–66)	16.055≠	0.000	S	0.004	0.000	0.500
	Range	12–110	12–96	10–147						

*P* value > 0.05, nonsignificant; *P* value < 0.05, significant; *P* value < 0.01,highly significant

*, Chi-square test; •, one-way ANOVA test

P1: Group A vs group B

P2: Group A vs group C

P3: Group B vs group C

## Discussion

Our study included 731 patients who presented to our university hospital to undergo elective coronary angiography for the diagnosis and treatment of CAD starting from September 2017 to August 2018. We aimed to evaluate the effect of prediabetes on angiographic outcomes in those patients. One hundred and seventy-seven patients were prediabetics constituting 24% of the study population. Similar prevalence of prediabetes was demonstrated among elective PCI patients and ACS patients in various registries [11, 12]. Patients with prediabetes had the same age range as diabetics and normoglycemic subjects, yet female gender was more prevalent among the diabetic group. This can be explained by the findings of Kodama et al. [13] suggesting that cardiovascular risk in the diabetic population is higher among women than in men. Although Kataoka et al. [14] and Choi et al. [11] found no significant difference in age between normoglycemic and prediabetic groups, the results of both showed male preponderance across the different groups. There were more smokers in the prediabetes group compared to diabetics (Choi et al.) [11]**.** However, we found that smoking was not significantly different between normoglycemic patients and prediabetics. There was a parallel increase in the prevalence of hypertension with increase in HBA1C. This can be attributed to insulin resistance promoting both hypertension and diabetes (Sowers) [15] or a myriad of genetic and environmental factors contributing to the development of both diabetes and hypertension [16]*.* Choi et al. [11] also demonstrated a higher prevalence of hypertension among prediabetic patients than normoglycemic patients undergoing elective PCI. Similarly, Zhang et al. [17] demonstrated that hypertension was more common in prediabetics than normoglycemic subjects and in diabetic group more than prediabetic group. Patients with prediabetes had a prevalence of dyslipidemia which was comparable to diabetics and normoglycemic subjects. Nakamura et al. [18] demonstrated that among CAD patients, prediabetics and diabetics showed a higher prevalence of dyslipidemia, yet this was evident in postprandial lipid levels and not the fasting lipid levels which are used as the standard screening test. Similarly, Açar et al. [12] found no difference in prevalence of dyslipidemia between prediabetic, normoglycemic, and diabetic subjects. The prevalence of CKD was not significantly different among the three groups, although diabetes is known as a common comorbid risk factor for CKD [19] as well as CKD pathophysiology starting in prediabetic subjects [20]. Those results are similar to Zhang et al. [17] and Choi et al. [11] who found no significant difference in prevalence of CKD among CAD patients. This can be attributed to hindering of both pharmacological and interventional treatment of cardiovascular disease by the presence of CKD in addition to increments in the risk of contrast-induced nephropathy with worsening of renal function; the management plan of CAD in CKD patients is directed towards more conservative management [21, 22]. Prediabetic subjects showed involvement of coronary arteries with a more aggressive atherosclerotic process resulting in CAD severity that was significantly higher than normoglycemic subjects and comparable to diabetic subjects. The number of coronary arteries with significant disease was higher in the prediabetic group than the normoglycemic group, yet there was no significant difference when compared with the diabetic group. This is similar to the findings of Santos et al. [23] who demonstrated that among patients with CAD confirmed by angiography, prediabetes was more commonly associated with multivessel disease. In addition, Açar et al. [12] found that among patients presenting with acute coronary syndrome, diabetic and prediabetic patients showed significantly higher prevalence of three vessel diseases when compared to normoglycemic patients. The complexity of CAD assessed by Gensini score was higher in the prediabetic than in normoglycemic subjects and comparable with diabetics. This is similar to the results of Açar et al. [12] where patients with prediabetes and diabetes showed a more complex coronary anatomy than normoglycemic subjects with a higher proportion of patients with three vessel diseases and higher CAD severity assessed by both SYNTAX and Gensini scores. This is in accordance with the results of Kataoka et al. [14]; both prediabetes group and diabetes group showed a higher Gensini score when compared to those without diabetes. The glycemic state didn’t affect the type of stent used, with drug-eluting stents (DESs) used in most of patients across the three groups. This goes hand in hand with Choi et al. [11] as all patients of the different groups received DESs. When comparing the length of stent used among the different groups, both prediabetics and diabetics required significantly longer stents than normoglycemic patients. This can be attributed to the findings of De Rosa et al. [24] who assessed plaque characteristics in stable CAD patients and demonstrated that both prediabetes and diabetes were associated with a higher and longer plaque burden. Zhang et al. [17] assessed OCT data regarding non-infarct-related plaques in patents presenting with ACS and found that raised HBA1C in prediabetic subjects was associated with more complex and active plaque structure with longer lipid length, higher lipid content, thinner fibrous cap, higher macrophage infiltration, wider lipid arc, and more calcification than normal subjects but was comparable to diabetic subjects. HBA1C was independently associated with significantly higher lipid length. Those results agree with the findings of Kataoka et al. [14] which demonstrated that both prediabetes and diabetes were associated with high average lesion length in patients with CAD assessed by quantitative coronary angiography. Similarly, Choi et al. [11] found significantly longer lesions in prediabetics when compared to normoglycemic subjects.

## Conclusion

Prediabetes is not merely a step closer to diabetes, it is a stage of diabetes which shows a similar atherosclerotic disease progression causing more complex coronary anatomy and requiring a higher number of longer stents. Yet, such a stage is always overlooked. Prediabetes confers high yet modifiable cardiovascular risk. Rigorous lifestyle interventions and medical treatment can help flatten the risk of conversion to diabetes, regression to normoglycemia, and reduction of the cardiovascular disease burden in this population.

## Data Availability

All data and equipment were available at Ain Shams University Ethics approval and consent to participate This study was approved by the Ethical Committee of Ain Shams university. All the procedures in the study were in accordance with the 1975 Helsinki Declaration, updated in 2013. Informed consent was obtained from all of the participants included in the study.

## Abbreviations

ACS: Acute coronary syndrome
ANOVA: Analysis of variance
BMS: Bare metal stent
CA: Coronary angiography
CABG: Coronary artery bypass grafting
CAD: Coronary artery disease
CKD: Chronic kidney disease
CVD: Cardiovascular disease
DES: Drug eluting stent
DM: Diabetes mellitus
eGFR: Estimated glomerular filtration rate
GFR: Glomerular filtration rate
HTN: Hypertension
IFG: Impaired fasting glucose
IGT: Impaired glucose tolerance
IQR: Interquartile range
LDL: Low-density lipoprotein
LM: Left main
MDRD: Modification of diet in renal disease
No.: Number
OCT: Optical coherence tomography
PCI: Percutaneous coronary intervention
SD: Standard deviation
